# Natural Variation Confers ‘Aiyuan 38’ Citrus Mutant a New Color and Unique Flavor

**DOI:** 10.3390/ijms24108816

**Published:** 2023-05-16

**Authors:** Tie Wang, Bo Xiong, Zhendong Zheng, Zeyu Qin, Lijun Deng, Wei Zheng, Mingfei Zhang, Guochao Sun, Siya He, Jun Wang, Zhihui Wang

**Affiliations:** College of Horticulture, Sichuan Agricultural University, Chengdu 611130, China; wangtie106@163.com (T.W.); xiongbo1221@sicau.edu.cn (B.X.); zzd530362810@163.com (Z.Z.); qinzeyuxx@163.com (Z.Q.); denglijun0919@163.com (L.D.); m18990820233@163.com (W.Z.); zhang_mingfei@sicau.edu.cn (M.Z.); sunguochao@163.com (G.S.); hesiya1114@163.com (S.H.); cnwangjun01@163.com (J.W.)

**Keywords:** citrus, bud mutation, flavor substances, HS-SPME-GC–MS, odor activity values

## Abstract

Citrus exhibits unique nutritional values. Most citrus cultivars are derived from mutations. However, the effect of these mutations on fruit quality is unclear. We have previously found a yellowish bud mutant in the citrus cultivar ‘Aiyuan 38’. Therefore, this study aimed to determine the effect of the mutation on fruit quality. ‘Aiyuan 38’ (WT) and a bud mutant variant (MT) were used to analyze variations in fruit color variation and flavor substances using colorimetric instruments, high-performance liquid chromatography (HPLC), headspace solid-phase microextraction-gas chromatography–mass spectrometry (HS-SPME-GC–MS), and odor activity values (OAVs). The mutation in MT conferred yellowish characteristics to its peel. Although the differences in total sugar and acid content of the pulp were not statistically significant between WT and MT, the MT glucose content was significantly lower and the malic acid level was significantly higher. HS-SPME-GC–MS analysis revealed that the MT pulp released more types and contents of volatile organic compounds (VOCs) than the WT, whereas the opposite trend was observed for the peel. Analysis of the OAV revealed that the MT pulp contains 6 unique VOCs, whereas the peel contains only 1. This study provides a useful reference for the study of flavor substances associated with citrus bud mutations.

## 1. Introduction

Citrus is a globally important horticultural crop with high nutritional value [1]. Citrus has become the most cultivated and productive fruit tree in tropical and subtropical regions [2]. Additionally, in agricultural production, most citrus cultivars were derived from spontaneous mutations [3]. Mutation breeding has become an important method for generating genetic variations and obtaining new varieties of several plants [4], including apples [5], citrus [6], plum [7], and kiwifruits [8]. Mutation breeding includes single nucleotide polymorphisms, a blend of insertions and deletions, translocations, inversions, and chromosomal mutations [9].

Recently, there has been increasing interest in the effect of mutations on fruit quality. Mutations in *PmUFGT3* (flavonoid-3-O-glycosyltransferase gene) result in peel color changes in Japanese apricots [10]. Mutations that cause color changes have also been observed in citrus [11], strawberry [12], and pepper [13]. Among the intrinsic fruit qualities, the components of flavor total soluble sugars (TSS) and titratable acids (TA) have become a hot research concern [14]. In orange mutational breeding, the mutants have significantly higher TSS/TA values compared to the wild type (WT), indicating that they would ripen slightly earlier than the parent plants, which affects their quality [15]. In addition, phenolic substances have good health benefits, and are quality indicators for breeding expeditions. In strawberry mutants, certain mutations confer higher total flavonoid content, especially of anthocyanins and proanthocyanidins, and combined transcriptional and metabolomic analyses revealed that these mutations alter the expression of some transcription factors (TFs) such as *C3H*, *MADS*, and *AP2*/*ERF* [16].

It is well known that the presence of volatile compounds and their composition determine the special aroma and flavor of food products, and related research has become a research hotspot [17]. Apple aroma volatile compounds, such as esters, are regulated by mutations [18]. For grapes, bud mutant fruits had a higher content of aromatic substances than the WT [19]. Interestingly, in chrysanthemum petal color mutants, the concentration of specific fragrance substances in the inflorescences is significantly different between the mutant and parent plants. The mutants contain two unique compounds: (S)-3-methylene-6-((R)-6-methylhept-5-en-2-yl) cyclohex-1-ene, and 4-methylene-1-(prop-1-en-2-yl) bicycle [3.1.0] hexane, indicating that the bud mutation confers a different fragrance to the flowers [20].

‘Aiyuan 38’ (*Citrus reticulata* ‘Aiyuan 38’), a hybrid between ‘Nanxiang’ and ‘Xizixiang’ available in November–January, is an excellent citrus variety. We have previously observed a yellowish bud mutant in ‘Aiyuan 38’ cultivars. Therefore, the aim of this study was to investigate the effect of bud mutation on fruit quality by comparing the differences in peel color between the WT and the mutant (MT), and by determining the intrinsic standard quality parameters of the pulp. Furthermore, we characterized the volatile organic compounds (VOCs) in the peel and pulp by headspace solid-phase microextraction-gas chromatography–mass spectrometry (HS-SPME-GC–MS), and analyzed the key aroma components using the OAV values. This study provides important insights into the effect of bud mutations on fruit quality, and lays a foundation for the certification and promotion of this variety.

## 2. Results and Discussion

### 2.1. Fruit Color Variation

**Figure 1:**
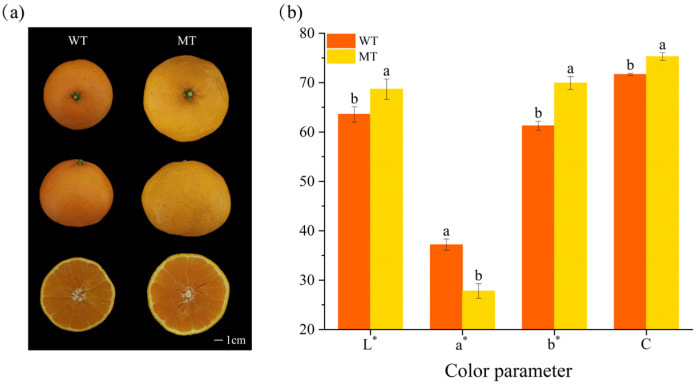
Fruit variation and color differences between WT and MT citrus. (**a**) Fruit appearance characteristics; (**b**) color parameter of the peel (L*, a*, b*, C). The different small letters on the bar graphs indicate significant differences (*p* < 0.05). WT, wild type; MT, mutant.

A clear difference in agronomic traits was observed between the WT and MT. The MT peel color had a notable yellowish hue compared to that of the WT (Figure 1a). To analyze the underlying mechanism of this color variation, we measured the color parameters using a CR-400 colorimeter. Significantly higher a* values were observed for the WT, whereas significantly higher values of L*, b*, and C were observed for the MT (Figure 1b). These results demonstrate that increasing values of L*, b*, and C could be the causative factors for the MT peel color variation. L*, a*, and b* are colorimetric systems developed by the Commission Internationale de l’Eclairage (CIE). In this system, three different characteristic parameters constitute the position of color in a three-dimensional color space. The L* indicates lightness ranging from 100 (white) to 0 (black), and a* and b* are color directions: −a* is the green axis, +a* is the red axis, −b* is the blue axis, and +b* is the yellow axis [21]. The C value indicates color purity, and the larger the value of C, the higher the purity and the more vivid the color [22]. Therefore, these results suggest that the bud mutation results in the MT peel possessing a significantly purer yellow hue.

### 2.2. Standard Quality Parameters

The fruit size, TA, and V_C_ were significantly different between the MT and WT (Table 1). Fruit size is an important agronomic parameter of fruit trees [23]. In the present study, we found that the weight of the MT fruit was 28% higher than that of the WT fruit and exhibited a significantly larger vertical/transverse diameter; however, the fruit shape index was similar. Mutations affecting fruit size have been previously reported in several studies [5,23]. The size increase is attributed to an increase in the number of cells, or a larger size of individual cells [24,25]; however, the exact mechanism requires further investigation.

TA and V_C_ are important factors in determining fruit quality. In our study, the TA content of the MT was significantly lower compared to that of the WT, whereas that of the TSS was not significantly different between the two plants, which may cause the MT to have a higher sweetness and adaptation to a wider population. V_C_ is a natural antioxidant with beneficial effects on human health [26]. In our study, the antioxidant capacity of the MT was reduced compared to that of the WT. The biological mechanisms governing V_C_ in plants are unclear, and it has been hypothesized that variations in V_C_ concentration are caused by either increased catabolism or decreased biosynthesis [27]. Overall, these results indicate that the MT had a larger fruit size and lower TA, whereas the WT had a higher antioxidant capacity.

### 2.3. Sugar and Acid Components

**Figure 2:**
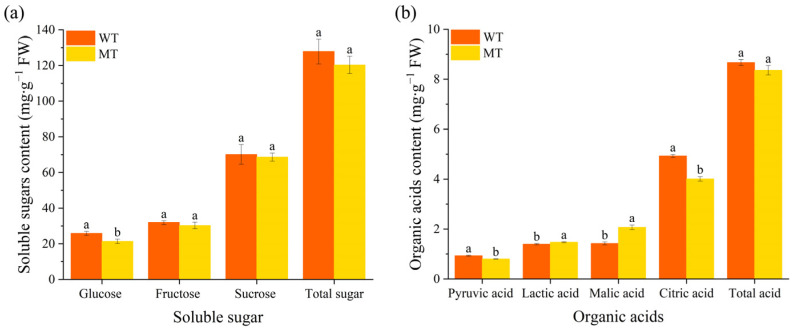
Sugar and acid content in fruits of WT and its varieties (MT). (**a**) Soluble sugar content; (**b**) organic acids content. The different small letters on the bar graphs indicate significant differences (*p* < 0.05). WT, wild type; MT, mutant.

Three types of soluble sugars (glucose, fructose, and sucrose) were detected in the pulp of WT and MT plants, with sucrose being the most abundant (Figure 2a). The glucose content in the MT was significantly lower than that in the WT; however, the other soluble sugar components and total sugar content were not significantly different. This result was consistent with the difference in the TSS content, suggesting that the mutation may have altered glucose production or metabolism in the MT pulp. Soluble sugars are important components of the flavor quality and nutritional composition of fruits, and various types of sugar components accumulate in fruits [28]. In the present study, the type and content of soluble sugars in the WT and MT were consistent, with sucrose being the highest in content, followed by fructose and glucose.

For all acid levels, the mean values for pyruvic, lactic, malic, and citric acids were significantly different between the WT and MT (Figure 2b). Citric acid had the highest accumulation in both plant types, followed by malic acid, which is consistent with previous studies [11,29,30]. Additionally, the WT had higher pyruvic acid and citric acid levels, whereas the MT had significantly higher lactic acid and malic acid levels. Citric acid gives citrus its acidity, and is an important component of citrus sensory quality [29]. In our study, citric acid levels were higher in the WT than in the MT, which explains the higher TA values (Table 1), because TA was determined as the equivalent amount of citric acid in 100 mL of juice.

### 2.4. Total Flavonoid and Phenolic Content

**Figure 3:**
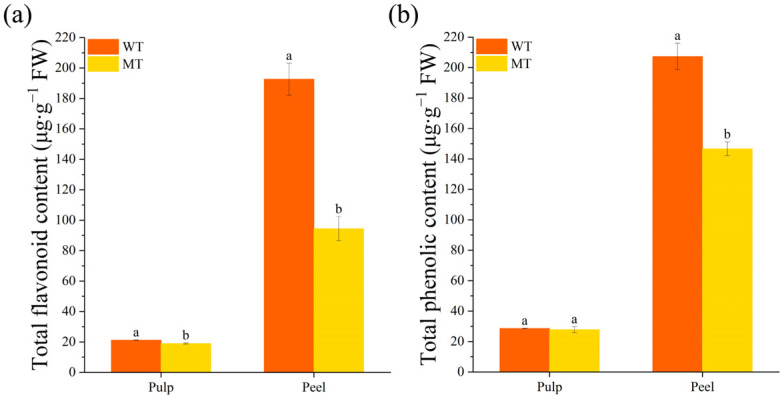
Total flavonoid and phenolic content in the pulp and peel of WT and MT. (**a**) Total flavonoid content; (**b**) total phenolic content. The different small letters on the bar graphs indicate significant differences (*p* < 0.05). WT, wild type; MT, mutant.

In addition to its high nutritional value, citrus fruit also has medicinal value. The flavonoids and phenolic compounds of citrus have strong antioxidant and anti-inflammatory activities in vitro and in vivo [31,32,33]. Therefore, we quantified the total flavonoid and phenolic contents in the pulp and peel of the WT and MT. The variation conferred lower total flavonoid and phenolic contents to the MT, especially in the peel (Figure 3a,b), indicating that the antioxidant capacity of the MT was lower than that of the WT.

Plant color is a complex regulatory system in which the specific color of a plant depends on auxiliary pigments, metal ions and pH, and the main color-developing pigments; among them, flavonoids are associated with the yellowish color trait of the plant [34]. Therefore, in the present study, the significant decrease in total flavonoids in the MT may be related to the variation in peel color (Figure 3a). This finding serves as a foundation for subsequent investigation of the causes of fruit peel color variation.

### 2.5. Correlation Analysis

**Figure 4:**
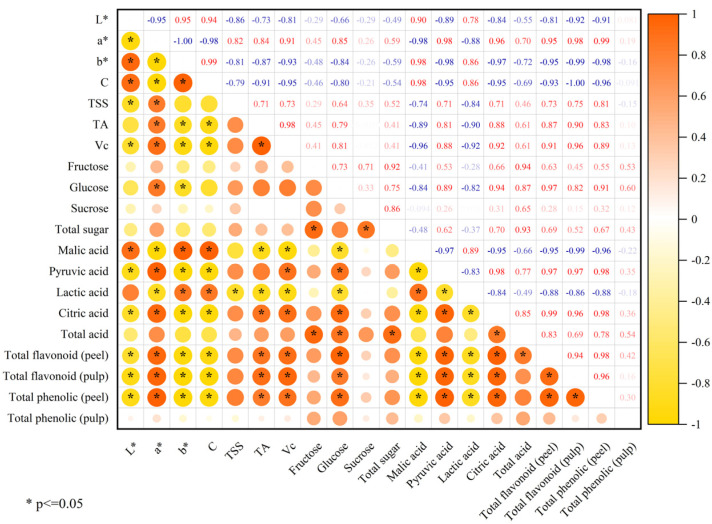
Correlation analysis of quality trait parameters.

We performed a correlation analysis of the quality trait parameters to investigate correlations between different indicators. (Figure 4). The total flavonoids in the peel were significantly negatively correlated with L*, b*, and C*, and significantly positively correlated with a*. This result further indicates that the variation in peel color was significantly correlated with the flavonoid content. However, the composition of peel color is a complex system, and its specific mechanisms need to be further explored. Additionally, the TA content was significantly and positively correlated with citric acid, which again suggests that citrus is dominated by the accumulation of citric acid.

### 2.6. VOCs

#### 2.6.1. Identification and Quantification of the VOCs in the Pulp of WT and MT

We identified 35 VOCs in the pulp of the WT and MT using HS-SPME-GC–MS (Table 2). D-Limonene, linalool B, and D-carvone were the main VOCs of WT and MT pulp. This result is consistent with the main citrus VOCs that have been previously reported [35,36,37].

In the present study, the total content of VOCs in the MT pulp was significantly higher than that in the WT pulp. In addition, the MT pulp had more types of VOCs (34) compared to those in the WT (25 VOCs). Ten VOCs, such as cosmene, decanal, and (1S,5S)-carvyl acetate, were identified only in the MT. Moreover, the MT pulp had higher amounts of alkenes, alcohols, ketones, aldehydes, and esters compared to the WT pulp. In conclusion, the richer fragrance of the MT pulp may be attributed to the higher content and various types of VOCs.

#### 2.6.2. Identification and Quantification of the VOCs in the Peel of WT and MT

As shown in Table 3, 69 and 66 VOCs were identified in the peels of the WT and MT samples, respectively. Trans-nerolidol, α-guaiene, and longipinene were only identified in the WT peel. Although there were significantly fewer VOCs in the MT peel than in the WT peel, there was significantly more (+)-4-carene and -cis-ocimene.

### 2.7. OAVs

**Figure 5:**
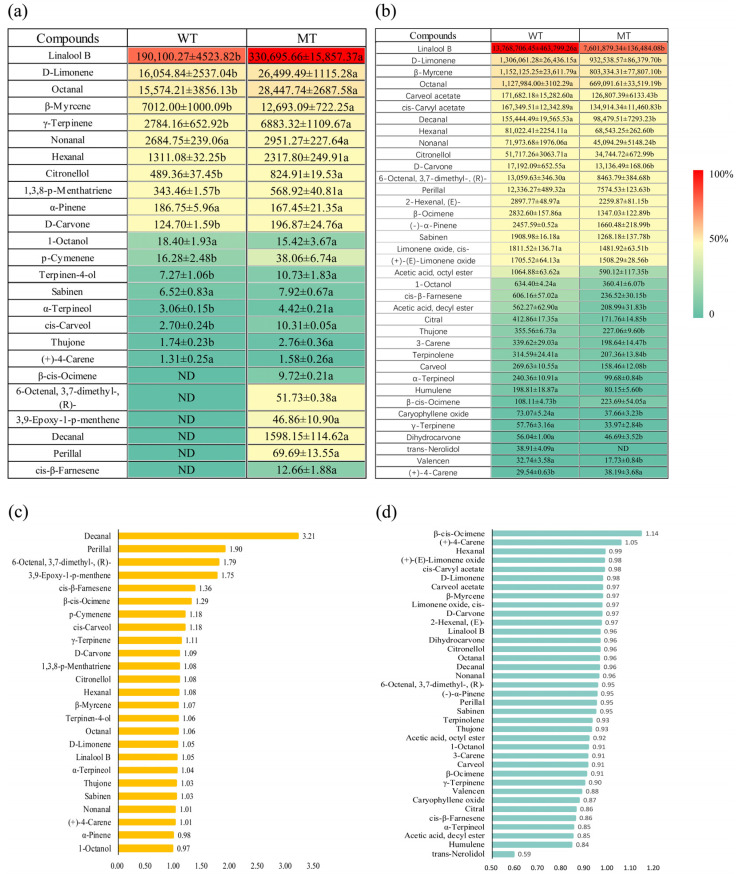
OAVs and normalization. (**a**) OAVs in the pulp; (**b**) OAVs in the peel; (**c**) normalized pulp OAVs; (**d**) normalized peel OAVs. OAV is the ratio of the concentration of the compound in the peel to the pulp to the threshold value in water; the different small letters after the numbers indicate significant differences (*p* < 0.05). OAV, odor activity value; WT, wild type; MT, mutant.

Aroma active substances are a class of substances that present a characteristic aroma, and the degree of contribution to the overall aroma of a sample can be assessed by OAVs [38,39]. In the present study, qualitative and quantitative analyses using HS-SPME-GC–MS were applied to calculate the OAVs of the plants, in combination with the threshold values of the characterized aromatic compounds (Figure 5). Olfactory thresholds of 19 and 25 VOCs found were characterized in the WT and MT pulp, respectively [40] (Figure 5a). Overall, the OAVs of all 25 substances in the MT pulp were significantly higher than those in the WT. β-cis-Ocimene, 6-octenal, 3,7-dimethyl-, (R)-, 3,9-epoxy-1-p-menthene, decanal, perillal, and cis-β-farnesene were only detected in the MT pulp. Decanal had the highest OAV, indicating it was unique to the MT and a high contributor of aroma. The odor profile of decanal is a sweet floral [41] and citrusy smell [42], and may play a role in the special aroma of MT.

In the peel, aroma thresholds were characterized for 37 VOCs in the WT and 36 VOCs in the MT (Figure 5b). The OAVs of all VOCs in the MT were considerably lower than those in the WT. However, the OAV of β-cis-ocimene and (+)-4-carene were considerably higher. Although there were differences in the types of aroma substances characterized in the peel and pulp, the highest contributors were linalool B and D-limonene, indicating that they were the main characteristic aromatic substances in the WT and MT. The presence of the above aromatic active compounds as major aromatic compounds in citrus was similar to results of other studies [43,44,45,46].

### 2.8. Normalization of OAVs

To compare the differences between the WT and MT OAVs, the results in Figure 5a, b were characterized using a normalized approach (Figure 5c,d). In the pulps, decanal, perillal, 6-octenal, 3,7-dimethyl-, (R)-, 3,9-epoxy-1-p-menthene, cis-β-farnesene, and β-cis-ocimene were significantly different between the two plant types (Figure 5c). In the peels, β-cis-ocimene was significantly different between the two plant types (Figure 5d). The MT peel and pulp had considerably more β-cis-ocimene than the WT, which could be attributed to the mutational variance of this variety.

## 3. Materials and Methods

### 3.1. Plant Materials and Treatment

‘Aiyuan 38’ yellowish bud mutation was found in Danling County, Meishan City, Sichuan Province in 2020, and the yellowish traits were observed to be stable on the parent plant and the heterozygous grafted plants. The experiment was conducted on fruit from mutant branches and fruit, while unmutated branches of the same plant were used as controls. The trees were 6 years old, tree management was carried out according to conventional management, and all stand conditions were basically the same. On 21 December 2021, 30 commercially mature WT and MT fruits were collected from the parent plant each, and three replications were set up with ten as one treatment. The collected fruits were quickly stored in ice boxes for rapid return to the laboratory. The vertical and transverse diameters of the fruits were measured with Vernier calipers, and an electronic balance was used to measure the weight of each fruit. The samples were frozen in liquid nitrogen immediately after separating the colored layer of the peel. One half of the pulp was immediately frozen in liquid nitrogen, and the other half was used for mass analysis. All frozen samples were stored at −80 °C until use.

### 3.2. Peel Color Measurement

The peel color was measured using a colorimeter (CR-400; Konica Minolta Inc., Tokyo, Japan). The color difference characteristics of six randomly chosen fruits from WT and MT plants were assessed along the equatorial surface of each fruit, with at least three replicates. The measured data included L*, a*, b* and C. The C was computed using the following formula: C = (a^2^ + b^2^)^1/2^ [22].

### 3.3. TSS, TA, and Vitamin C (V_C_)

The TSS and TA contents were determined using an integrated sugar and acid machine (Pocket PAL-BXIACID1; ATAGO, Tokyo, Japan). After zeroing the machine with distilled water, 0.3 mL of juice was added into the measuring port of the machine, and the START button was pressed to measure the TSS. Then, to 1 mL of juice was added 50 mL of distilled water for dilution, the measurement mode of the machine was adjusted, and the same method was used for TA measurement. The V_C_ content was determined using the method described by Kelebek and Selli with minor modifications [47]. A 5 mL volume of juice was diluted with 1% oxalic acid to fix the volume to 50 mL. The dilution was pipetted 5 mL at a time into a 50 mL triangular flask by titrating with 2, 6-dichlorophenol indophenol dye solution until a stable light pink color was obtained.

### 3.4. Sugar and Acid Compounds

The sugar and acid components of the pulp were measured using high performance liquid chromatography (HPLC). The 2 g of mixed pulp was accurately weighed, and 4 mL of distilled water was added; the mixture was shaken well and placed in an 80 °C water bath for 15 min. After cooling at room temperature, the mixture was run in a centrifuge at 4 °C at 9000 r/min for 15 min; the supernatant was taken, and the residue was centrifuged again with 4 mL of distilled water at the same conditions for 15 min. The supernatant was taken and fixed to a volume of 10 mL. The sample was then transferred by syringe through a 0.45 μm aqueous phase filter membrane to the injection vial for analysis of the sugar components [48]. The mixed pulp samples were weighed to 0.5 g, ground with pre-chilled 3 mL of 0.2% metaphosphoric acid, fixed to 6 mL, and centrifuged at 4 °C for 15 min at 12,000 r/min. The supernatant was extracted and filtered through a 0.22 μm aqueous membrane for the determination of organic acid components [49,50]. The content of the sugars and acids were determined using the standard curve for each substance. Three biological replicates were taken for all indicators, and technical replicates were performed three times.

### 3.5. Total Flavonoid and Phenolic Contents

The total flavonoid contents were determined by a colorimetric method with aluminum chloride [51]. The extract was mixed with 75 μL of 95% ethanol, 10 μL of 10% aluminum chloride, 10 μL of 1.0 M potassium acetate, and 140 μL of distilled water. The absorbance of the reaction mixture against the blank was measured at 415 nm after incubation for 40 min at room temperature (27 ± 1 °C). A standard curve was made with quercetin (0–250 μg/mL), and the data units are μg g^−1^ FW. The Folin–Ciocalteu method was used to quantify the total phenolic contents [52]. Briefly, 12.5 μL of the water-soluble extract was mixed with 250 μL of 2% sodium carbonate solution and reacted for 5 min at room temperature. Then, 12.5 μL of Folin–Ciocalteu phenol reagent (50%) was added and allowed to stand for 30 min at room temperature, and then the absorbance of the reaction mixture was read at 650 nm. The standard curves were made with gallic acid aqueous solution (100–1000 μg/mL), and the data units are μg g^−1^ FW.

### 3.6. VOC Identification and Quantification

HS-SPME-GC–MS was used to extract and detect VOCs in different parts of the WT and MT fruits. Fruit tissues (0.1 g peel and 0.5 g pulp) were ground into powder using liquid nitrogen, and the VOCs were then measured. The sample was weighed and quickly placed into a 20 mL headspace flask; 5 mL of saturated sodium chloride solution was added, and then 50 μL of 1-hexanol (0.1%, *v/v*) was added as an internal standard. The solution was mixed well. After equilibration at 42 °C for 30 min, extraction was performed using a 50/30 μm divinylbenzene/carboxen/polydimethylsiloxane (DVB/CAR/PDMS) extraction head (Supelco Co., Bellefonte, PA, USA) for 30 min. The extraction head was further inserted into a GC–MS instrument (7890; Agilent Technologies, Santa Clara, CA, USA) for desorption. The desorption was performed in a non-split mode at 250 °C for 5 min on an HP-5MS column (30 m × 0.25 mm × 0.25 um, J&W Scientific, Folsom, CA, USA), with a carrier gas of 1.0 mL·min^−1^ He and an oven temperature ramp-up program of 40 °C for 3 min, 3 °C·min^−1^ to 70 °C, 1 °C·min^−1^ to 130 °C, and 15 °C·min^−1^ to 230 °C. The MS conditions were set to 230 °C for the ion source and 70 eV for the electron energy [53].

The data collected from GC–MS were processed with the Data Analysis Application (www.agilent.com/chem (accessed on 27 October 2022), and VOCs were identified with the mass spectrometry library NIST-11.0/14.0 (NIST/EPA/NIH, USA) in combination with substance retention indices (RIs). The RIs of the VOCs were calculated from a series of n-alkanes (Alkane Mix 34, C7-C40; ANPEL Laboratory Technologies, Shanghai, China), and the calculation formula used was based on a previous study [54]. The VOC concentrations were calculated using the area normalization method, and the substances were categorized according to the previous method [55].

### 3.7. Odor Activity Value (OAV) Calculation and Normalization

The OAVs were calculated according to the following formula: OAV = Ci/Ti, where Ci is the defined concentration of compound i, and Ti is its odor threshold value in water [56]. All of the thresholds used in this study were obtained from a previously published study [40]. The content of peel and pulp OAVs was normalized in Excel (ver. 2016) using the formula LOG((MT + 10),2)/LOG((WT + 10),2), with WT as the control.

### 3.8. Statistical Analysis

The experimental data were analyzed using an independent samples t-test with IBM SPSS Statistics 23.0 (IBM, Armonk, NY, USA). Differences between the samples were tested using Duncan’s multiple-range test. The statistical significance was set at *p* < 0.05. A correlation analysis was performed using Origin 2021 software (OriginLab Corporation, Northampton, MA, USA).

## 4. Conclusions

In summary, this study analyzed the differences in color and flavor substances between WT and MT fruits using colorimetric instruments, HPLC, HS-SPME-GC–MS, and OAV techniques. We revealed that bud mutation conferred yellowish color characteristics to the WT peel, which is presumably related to the change in total flavonoid content. The MT pulp contained lower V_C_, glucose, pyruvate, and citric acid, but significantly higher levels of lactic and malic acid. Additionally, the MT pulp contained higher VOC content and more VOC species than the WT pulp; however, the opposite was observed for the peel. We screened 6 unique OAVs in the MT pulp: decanal, perillal, 6-octenal, 3,7-dimethyl-, (R)-, 3,9-epoxy-1-p-menthene, cis-β-farnesene, and β-cis-ocimene. The MT peel had a significantly higher β-cis-ocimene content compared to the WT peel. Therefore, further studies should focus on verifying the effects of bud mutations on β-cis-ocimene production.

## Figures and Tables

**Table 1:** Comparison of fruit quality of Aiyuan 38 (WT) and its varieties (MT).

Fruit Type	Fruit Weight (g)	Vertical Diameter (mm)	Transverse Diameter (mm)	Fruit Shape Index	TSS (%)	TA (%)	TSS/TA Ratio	V_C_ (mg/100 mL FW)
WT	174.73 ± 5.09 b	61.76 ± 1.93 b	70.96 ± 1.32 b	0.87 ± 0.03 a	9.07 ± 0.06 a	0.92 ± 0.01 a	9.88 ± 0.12 a	30.37 ± 0.56 a
MT	223.70 ± 20.90 a	68.42 ± 3.23 a	77.75 ± 2.91 a	0.88 ± 0.05 a	8.97 ± 0.06 a	0.89 ± 0.01 b	10.08 ± 0.08 a	27.91 ± 0.54 b

TSS, total soluble sugars; TA, titratable acid; V_C_, vitamin C. The different small letters indicate significant differences (*p* < 0.05).

**Table 2:** Identification and quantification of the volatile organic compounds in the pulp of WT and MT citrus (μg/g).

Compound Names	RI	WT	MT
**Esters**			
cis-Carvyl acetate	1336	1.10 ± 0.12 b	4.12 ± 0.66 a
(1S,5S)-Carvyl acetate	1363	ND	4.20 ± 0.12 a
Subtotal		1.10 ± 0.12 b	8.32 ± 0.60 a
**Alcohols**			
1-Octanol	1148	2.32 ± 0.24 a	1.94 ± 0.46 a
Linalool B	1099	41.82 ± 1.00 b	72.75 ± 3.49 a
cis-p-Mentha-2,8-dien-1-ol	1139	4.52 ± 0.17 b	7.67 ± 0.38 a
Terpinen-4-ol	1194	8.72 ± 1.27 b	12.87 ± 2.20 a
α-Terpineol	1215	3.68 ± 0.18 b	5.31 ± 0.24 a
cis-Carveol	1257	0.68 ± 0.06 b	2.58 ± 0.01 a
Citronellol	1280	3.91 ± 0.30 b	6.60 ± 0.16 a
Subtotal		65.65 ± 1.52 b	109.72 ± 0.10 a
**Ketones**			
Thujone	1115	0.63 ± 0.08 b	0.99 ± 0.13 a
(+)-2-Bornanone	1147	ND	0.94 ± 0.09 a
D-Dihydrocarvone	1223	1.13 ± 0.18 b	1.86 ± 0.17 a
D-Carvone	1294	19.95 ± 0.25 b	31.50 ± 3.96 a
cis-Geranylacetone	1450	1.08 ± 0.04 a	1.19 ± 0.20 a
Subtotal		22.61 ± 0.65 b	36.48 ± 4.38 a
**Aldehydes**			
Hexanal	810	6.56 ± 0.16 b	11.59 ± 1.25 a
Octanal	1033	7.79 ± 1.93 b	14.22 ± 1.34 a
Nonanal	1141	2.95 ± 0.26 a	3.25 ± 0.25 a
6-Octenal, 3,7-dimethyl-, (R)-	1166	ND	1.55 ± 0.01 a
Decanal	1243	ND	4.79 ± 0.34 a
Perillal	1342	ND	2.09 ± 0.41 a
Subtotal		17.30 ± 2.06 b	37.50 ± 3.15 a
**Alkenes**			
α-Pinene	949	2.61 ± 0.08 a	2.34 ± 0.30 a
Sabinen	978	6.39 ± 0.81 a	7.76 ± 0.65 a
β-Myrcene	993	8.41 ± 1.20 b	15.23 ± 0.87 a
(+)-4-Carene	1013	1.31 ± 0.24 a	1.58 ± 0.26 a
D-Limonene	1025	545.86 ± 86.26 b	900.98 ± 37.92 a
β-cis-Ocimene	1044	ND	0.53 ± 0.01 a
γ-Terpinene	1052	0.72 ± 0.17 b	1.78 ± 0.29 a
p-Cymenene	1084	1.38 ± 0.21 b	3.24 ± 0.57 a
1,3,8-p-Menthatriene	1120	5.15 ± 0.02 b	8.53 ± 0.61 a
Cosmene	1229	ND	8.35 ± 0.67 a
2,6-Octadiene, 2,6-dimethyl-	1493	ND	3.14 ± 0.14 a
cis-β-Farnesene	1450	ND	2.03 ± 0.30 a
Valencen	1477	3.49 ± 0.50 a	3.96 ± 0.88 a
Subtotal		575.34 ± 87.02 b	959.46 ± 41.11 a
**Others**			
3,9-Epoxy-1-p-menthene	1206	ND	1.41 ± 0.33 a
3-Cyclohexen-1-ol, 5-methylene-6-(1-methylethenyl)-	1210	4.25 ± 0.68 a	ND
Subtotal		4.25 ± 0.68 a	1.41 ± 0.33 b
Total		686.43 ± 86.05 b	1152.89 ± 48.38 a

Different small letters indicate significant differences (*p* < 0.05). RI, retention index; MT, mutant; WT, wild type; ND, not detected.

**Table 3:** Identification and quantification of the volatile organic compounds in the peel of WT and MT citrus (μg/g).

Compound Names	RI	WT	MT
**Esters**			
Acetic acid, octyl ester	1257	50.05 ± 2.99 a	27.74 ± 5.52 b
Myrtenyl acetate	1321	21.76 ± 3.74 a	12.03 ± 0.90 b
cis-Carvyl acetate	1337	251.02 ± 18.52 a	202.37 ± 17.19 b
Carveol acetate	1363	257.52 ± 22.93 a	190.21 ± 9.20 b
Acetic acid, decyl ester	1421	22.49 ± 2.52 a	8.36 ± 1.27 b
Subtotal		602.85 ± 47.68 a	440.71 ± 29.40 b
**Alcohols**			
cis-4-Thujanol	1062	102.86 ± 9.78 a	47.87 ± 3.05 b
1-Octanol	1148	79.81 ± 0.54 a	45.34 ± 0.76 b
Linalool B	1104	3029.12 ± 102.04 a	1672.41 ± 30.03 b
(E)-p-2,8-Menthadien-1-ol	1122	101.26 ± 1.91 a	39.46 ± 1.65 b
α-Terpineol	1216	288.43 ± 13.09 a	119.62 ± 1.01 b
cis-Carveol	1261	125.32 ± 15.76 a	61.50 ± 0.06 b
Carveol	1280	67.41 ± 2.64 a	39.62 ± 3.02 b
Citronellol	1285	413.74 ± 24.51 a	277.96 ± 5.39 b
trans-Nerolidol	1562	9.73 ± 1.03 a	ND
Spathulenol	1564	41.32 ± 3.86 a	23.50 ± 2.74 b
Subtotal		4258.98 ± 172.74 a	2327.27 ± 38.17 b
**Ketones**			
Thujone	1116	128.00 ± 2.42 a	81.74 ± 3.46 b
Pinocarvone	1173	11.63 ± 0.15 a	6.10 ± 0.33 b
trans-3-Pinanone	1188	50.07 ± 4.52 a	32.56 ± 2.12 b
Dihydrocarvone	1223	182.12 ± 3.26 a	151.74 ± 11.44 b
D-Carvone	1300	2750.73 ± 104.41 a	2101.84 ± 26.89 b
Subtotal		3122.55 ± 105.71 a	2373.98 ± 43.21 b
**Aldehydes**			
Hexanal	782	405.11 ± 11.27 a	342.72 ± 1.32 b
2-Hexenal, (E)-	874	257.03 ± 4.35 a	200.45 ± 7.20 b
Octanal	1037	563.99 ± 1.55 a	334.55 ± 16.76 b
Nonanal	1144	79.17 ± 2.17 a	49.61 ± 5.67 b
6-Octenal, 3,7-dimethyl-, (R)-	1167	391.79 ± 10.39 a	253.91 ± 11.54 b
Decanal	1244	466.33 ± 58.70 a	295.44 ± 21.88 b
Perillal	1342	370.09 ± 14.68 a	227.24 ± 3.71 b
Citral	1345	49.54 ± 2.08 a	20.61 ± 1.78 b
Subtotal		2583.06 ± 93.40 a	1724.52 ± 63.39 b
**Alkenes**			
β-Thujene	946	65.86 ± 2.46 a	40.43 ± 3.91 b
(-)-α-Pinene	949	245.76 ± 0.05 a	166.05 ± 21.90 b
Sabinen	979	1870.80 ± 15.86 a	1242.81 ± 135.03 b
β-Myrcene	995	1382.55 ± 28.33 a	964.00 ± 93.37 b
(+)-4-Carene	1018	29.54 ± 0.63 b	38.19 ± 3.68 a
D-Limonene	1037	44,406.08 ± 898.83 a	31,706.31 ± 2936.91 b
β-cis-Ocimene	1038	5.95 ± 0.26 b	12.30 ± 2.98 a
β-Ocimene	1046	96.31 ± 5.37 a	45.80 ± 4.18 b
γ-Terpinene	1054	57.76 ± 3.16 a	33.97 ± 2.85 b
Terpinolene	1084	62.92 ± 4.88 a	41.47 ± 2.77 b
Cosmene	1110	9.77 ± 0.64 a	5.10 ± 0.47 b
Limonene oxide, cis-	1137	452.88 ± 34.18 a	370.48 ± 15.88 b
cis-p-Mentha-2,8-dien-1-ol	1140	116.42 ± 4.04 a	39.45 ± 0.84 b
(+)-(E)-Limonene oxide	1143	426.38 ± 16.03 a	377.07 ± 7.14 b
Camphene	1238	28.80 ± 1.05 a	11.31 ± 0.39 b
α-Cubebene	1330	31.73 ± 3.37 a	13.80 ± 1.39 b
2,6-Octadiene, 2,6-dimethyl-	1494	337.99 ± 29.39 a	227.06 ± 15.49 b
3-Carene	1513	261.51 ± 22.36 a	152.95 ± 11.15 b
β-Cubebene	1371	60.45 ± 4.61 a	29.72 ± 3.79 b
β-Elemene	1374	77.00 ± 7.89 a	37.20 ± 2.16 b
4-Tetradecene, (E)-	1382	13.86 ± 1.42 a	6.21 ± 0.99 b
Zingiberene	1393	78.20 ± 7.88 a	35.54 ± 3.63 b
Aromandendrene	1418	10.40 ± 1.54 a	2.83 ± 0.36 b
α-Guaiene	1422	4.62 ± 0.17 a	ND
Humulene	1433	31.81 ± 3.02 a	12.82 ± 0.90 b
cis-β-Farnesene	1445	96.99 ± 9.13 a	37.84 ± 4.83 b
(E)-β-Famesene	1452	1286.95 ± 122.95 a	524.50 ± 17.50 b
β-Copaene	1464	26.75 ± 3.26 a	8.02 ± 0.53 b
Longipinene	1470	12.43 ± 1.38 a	ND
Curcumene	1474	27.38 ± 2.57 a	12.49 ± 0.84 b
Valencen	1478	343.80 ± 37.64 a	186.18 ± 8.84 b
Bicyclogermacrene	1481	77.20 ± 14.63 a	35.78 ± 2.93 b
α-Muurolene	1488	25.44 ± 3.06 a	13.70 ± 1.09 b
α-Bulnesene	1492	9.24 ± 0.70 a	4.49 ± 0.30 b
Pentadecane	1499	6.01 ± 0.60 a	4.64 ± 0.18 b
γ-Muurolene	1501	42.09 ± 4.28 a	17.29 ± 0.12 b
trans-Calamenene	1511	11.04 ± 0.05 a	4.07 ± 0.67 b
δ-Cadinene	1513	104.62 ± 10.18 a	40.58 ± 5.42 b
Sesquiphellandrene	1516	54.70 ± 5.69 a	15.57 ± 2.66 b
α-Farnesene	1523	7.42 ± 0.25 a	4.19 ± 0.22 b
Caryophyllene oxide	1568	29.96 ± 2.15 a	15.44 ± 1.32 b
Subtotal		52,327.33 ± 1133.87 a	36,537.65 ± 3204.70 b
Total		62,894.77 ± 1535.96 a	43,404.12 ± 3330.26 b

Different small letters indicate significant differences (*p* < 0.05); RI, retention index; MT, mutant; WT, wild type; ND, not detected.

## Data Availability

Data are contained within the article.
